# Physical Obstruction of Nasal Cavities With Subsequent Asphyxia, Causes Lethality of Rats in an Acute Inhalation Study With Hydrophobic HMDZ Surface-Treated Synthetic Amorphous Silica (SAS)

**DOI:** 10.3389/fpubh.2022.907078

**Published:** 2022-06-03

**Authors:** Nils Krueger, Klaus Weber, Nils Warfving, Alex Vitali, Jürgen Nolde, Tobias B. Schuster, Gustav Gerd Bruer, Otto Creutzenberg, Benno Wessely, Michael Stintz, Valerie Moise, Marco Kellert

**Affiliations:** ^1^Evonik Operations GmbH, Smart Materials, Hanau, Germany; ^2^AnaPath Services GmbH, Liestal, Switzerland; ^3^Grace Europe Holding GmbH, Worms, Germany; ^4^Department of Inhalation Toxicology, Fraunhofer Institute for Toxicology and Experimental Medicine (Fh-ITEM), Hannover, Germany; ^5^Institute of Process Engineering and Environmental Technology, Research Group Mechanical Process Engineering, Technische Universität Dresden, Dresden, Germany; ^6^Cabot Corporation, Corporate SHE - Product Safety and Toxicology, Loncin, Belgium; ^7^Wacker Chemie AG, Burghausen, Germany

**Keywords:** synthetic amorphous silica, inhalation, rat, contact angle, physical obstruction, suffocation, asphyxia

## Abstract

The aim of the present study was to understand the mechanism of lethality associated with high dose inhalation of a low-density hydrophobic surface-treated SAS observed in some acute inhalation studies. It was demonstrated that physical obstruction of the upper respiratory tract (nasal cavities) caused the effects observed. Hydrophobic surface-treated SAS was inhaled (flow-past, nose-only) by six Wistar rats (three males and three females) in an acute toxicity study at a concentration of ~500 mg/m^3^ for an intended 4-hr exposure. Under the conditions of the test set-up, the concentration applied was found to be the highest that can be delivered to the test animal port without significant alteration of the aerosol size distribution over time. None of the test- material-exposed animals survived the planned observation time of 4 h; three animals died between 2$\frac{3}{4}$ h after starting exposure and cessation of exposure at 3$\frac{1}{4}$ h, two died after transfer to their cages and the remaining animal was sacrificed due to its poor condition and welfare considerations. Histology accomplished by energy dispersive X-ray (EDX) analysis demonstrated that test material particles agglomerated and formed a gel-like substrate that ultimately blocked the upper respiratory airways, which proved fatal for the rat as an obligatory nose breather. This observation is in line with the findings reported by Hofmann et al. showing a correlation between lethality and hydrophobicity determined by contact angle measurement. The aerosol characterizations associated with this study are provided in detail by Wessely et al.

## Introduction

Particles with low systemic toxicity such as titanium dioxide (TiO_2_) or different forms of synthetic amorphous silica (e.g., SAS, SiO_2_) are currently under regulatory scrutiny. The classification of certain forms of titanium dioxide (TiO_2_) as suspected carcinogens by inhalation was published on February 2, 2020 amending the EC regulation no. 1272/2008 (CLP Regulation). TiO_2_ as E 171 was removed from the EU list of approved food additives [Com Reg (EU) 2022/63] based on a scientific opinion by the European Food Safety Authority (EFSA). The origin of this regulatory pressure is partly due to the particles falling under the nanomaterial definition but also to guidance values for classification and labeling that have never been validated for the local respiratory effects caused by particles. Strictly following the CLP guidance would result in classifications for these particles based on the animal toxicity studies; however, it is important to consider whether the substance form reflects exposures for humans. The particles of these and other substances have in common a low bulk density (according to TRGS 527) and no systemic toxicity but, are either already classified, or in the process of being classified as ‘a hazardous or toxic substances' under CLP regulation in the EU with severe consequences for production processes and applications, whereas TRGS 527 is suggesting an occupational exposure limit (OEL) as an alternative.

Low-density particles are extremely light and fluffy. This not only means that on a mass basis there are many more particles compared to a standard dust aerosol, but also that the quite large fluffy aggregates and agglomerates would result in easier blockage of the nasal cavities in the rat compared to standard dust particles. Therefore, high concentrations of low-density particles, such as hydrophobic SAS, can cause lethality in acute inhalation tests at concentrations <1,000 mg/m^3^ [ECETOC JACC Report No. 51, (1)]. In OECD guidance 403 and 436 studies (2, 3), observed lethality below 1,000 mg/m^3^ within 4 h can lead to skull and crossbones labeling (toxic by inhalation) with respect to the CLP classification and labeling criteria. Recently, the European Risk Assessment Committee (RAC) proposed the Acute Tox Cat 2 (with a hazard statement: “Fatal if inhaled”) classification for HMDZ (hexamethyldisilazane), a surface-treated SAS, and one form of hydrophobic SAS, based on the lethality in test animals observed within 4 h at 540 mg/m^3^ in an acute inhalation study with read-across to hydrophobic DDS (dimethyldichlorosilane) surface-treated SAS showing a calculated LC50 of 450 mg/m^3^ (4). No mortalities were observed with this substance in the low-concentration group (210 mg/m^3^), seven of 10 died in the mid-concentration group (540 mg/m^3^) and all the rats died (100% mortality) in the high-concentration group (2,100 mg/m^3^). The calculated LC50 of 450 mg/m^3^ is generally an extremely high concentration for particles, particularly for SAS when taking into account that the occupational exposure limit (OEL) for SAS in the German TRGS 900 is 4 mg/m^3^ inhalable which is more than 100 times lower. Furthermore, amorphous silica is not associated with any intrinsic toxicity. SAS forms are used in food, feed and cosmetic applications. Lindner et al. (5) were able to show in their study that nanostructured biogenic amorphous silica (BAS) occurs in food products such as common horsetail and oat husk and that electron microscopical examination showed no morphological differences from synthetic amorphous silica. *In vitro* studies performed by Wiemann et al. (6) showed that surface treatment with hydrophobic coating reagents (organosilanes) strongly reduces the bioactivity of SAS. Against this background, the question arises as to what could cause HMDZ surface-treated SAS lethality in acute inhalation studies. Is it systemic toxicity of the substance or is it more likely that, based on the high concentrations applied in acute inhalation studies, we are dealing with a high-dose phenomenon associated with physical obstruction of the upper respiratory tract? Physical obstruction at high particle concentrations is considered in OECD guidance 39 (7); paragraph [69] explicitly states that “*At very high concentrations, dry powder aerosols... tend to form conglomerates in the proximal nose causing physical obstruction of the animals' airways (e.g., dust loading) and impaired respiration which may be misdiagnosed as a toxic effect.”*

Regarding the objective of clarifying the cause of lethality associated with physical obstruction of the upper respiratory tract, it needs to be clearly stated that the studies carried out under the OECD guidelines cannot answer this question. In cases of lethality, OECD 403 and 436 studies for acute inhalation require a dead animal count and only a cursory macroscopical examination of the outer surfaces of the organs in the abdominal and thoracal cavity. Thorough pathological and histopathological examinations of the entire respiratory tract, especially the upper respiratory tract (nasal cavities) is not required. These OECD guidelines are fully sufficient to identify general systemic effects but specific particle- related questions on fatal local activity cannot be answered, e.g., the physical obstruction of the upper respiratory tract leading to suffocation as cause of mortality. Rat respiratory tract anatomy differs to that of humans: there is mainly monopodial branching of airways, smaller ventilatory unit volume, smaller alveolar size and a lower average number of cells per alveolus (8). Unlike to humans, the rat is an obligatory nose breather; while fixed in a tube for 4 h during acute inhalation studies, the rat can neither protect its nose nor can it even carry out normal cleaning behavior. Taking all these points into account, a new GLP acute inhalation study with HMDZ surface-treated SAS was performed to allow possible areas of obstruction to be identified. Additional parameters were considered in the design of this new study (Table 1). A thorough histopathological examination of the entire respiratory tract, including different levels of the nasal cavities, larynx, trachea and lung was included and EDX analysis was performed to determine the chemical identity of agglomerated material. To characterize hydrophobicity of the tested substance, contact angle measurements were made. Aerosol generation considerations are the subject of a separate publication (9). To assure the validity of this new acute inhalation toxicity study with HMDZ surface-treated hydrophobic SAS, it was necessary to select the highest technically feasible concentrations without significant aerosol alteration.

**Table 1:** Extended experimental design of the acute inhalation test.

1	To take four-chamber aerosol samples during a 4 h study (gravimetrical filter analysis) at an outlet port of the nose-only platform; the aerosol concentrations 50, 500, 1,000, and 2,000 mg/m^3^ required by OECD TG 436 will be adjusted to use a target concentration of 500 mg/m^3^ as a starting point; the exact value may increase during the 4 h exposure (saturation effect of the exposure unit). *Due to dead/moribund animals, the exposure was stopped after 3 h and surviving animals were transferred to cages. Because of the shortened exposure period, only three gravimetrical filters were taken. 494.0, 519.8, and 537.9 mg/m^3^; mean: 517.2 ± 18.0 mg/m^3^ (N = 3). An on-line recording of the aerosol concentration was performed in parallel using an aerosol photometer*.
2	To determine the particle size distribution at least twice during a 4 h study; cascade impactor; aerosol generation must be established and controlled over 4 h to avoid too strong agglomerate formation at the outlet ports of the nose-only platform. *Only one value determined because of the shortened exposure period: MMAD = 1.31 μm*
3	To record body weights during the day 1 and 14 post-exposure observation periods.
4	To record gross pathological changes of those rats that died or were killed in a moribund condition prior to the end of the day 1 and 14 post-exposure observation periods.
5	To record gross pathological changes of all animals at terminal sacrifice.
6	To characterize hydrophobicity of the tested substance contact angle measurements were conducted.
7	To histopathologically examine the entire respiratory tract of the animals at days 1 and 14 post-exposure including different levels of the nasal cavities, larynx, trachea and lung. Histopathology was performed in combination with EDX analysis to determine chemical identity of agglomerated material. *Due to dead/moribund animals, the exposure was stopped after 3 h and histopathological as well as EDX examinations were performed for day 1 only*.
8	All observations helping to explain the potential cause of mortality will be documented. This includes photographs, especially of the external nose area (nostrils and whiskers) during and after exposure, to make sure that important details, such as a clot of particulate material at the tip of the nose, are documented (the rat is an obligatory nose breather).

Considering the rat nose, the mass median aerodynamic diameter (MMAD) of the aerosol had to fulfill the ≤4 μm criterion. The test concentration in this study was 500 mg/m^3^.based on the investigations from Wessely et al. (9).

## Materials and Methods

### Extended Study Design

Table 1 lists the parameters that were used in addition to those indicated in OECD TG 436 as minimum obligatory requirements for a regulatory test of acute toxicity following inhalation.

### Test Item

HMDZ surface-treated SAS (CAS number: 68909-20-6; CAS name: *Silanamine, 1,1,1- trimethyl-N-(trimethylsilyl)-, hydrolysis products with silica*), lot #150060338, was provided by Evonik Operations GmbH, Germany. The test material exhibits the following physicochemical properties: BET: 230–290 m^2^/g (medium surface area), purity 99.8% (based on ignited material), delivered as fluffy, white powder with a skeletal density of 2.3 g/cm^3^ at 20°C by helium pycnometry (3P Instruments) and a tamped density of about 0.06 g/cm^3^.

### Test Item Characterization and Optimization

**Figure 1:**
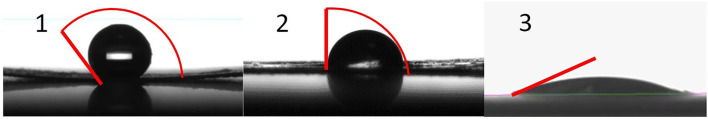
Contact angle for different synthetic amorphous silica products, (1) HMDZ surface- treated SAS, (2) untreated pyrogenic silica, (3) untreated silica gel (10).

Figure 1 shows the contact angle of the test material as measured according to DIN 55660-2 (11) and Hofmann et al. (12). The measurement was performed using a contact angle instrument, the DSA100 Drop Shape Analyzer and ADVANCE software (KRÜSS Scientific, Germany).

### Aerosol Generation

The rats were exposed to an aerosol generated by a flow-past, nose-only inhalation exposure system which has been used for previous inhalation studies at Fraunhofer ITEM (13). In this system, the animal's snout is placed in the anterior end of the tube, which is connected to the exposure cylinder by means of a push fit. The aerosols enter continuously the animal's nasal region through a small tube. The exposure cylinder is operated at slightly positive pressure with respect to the surrounding air. This ensures that a continuous air flow is passing through the animal's breathing zone. In this system, the aerosol is supplied to each rat individually and exhaled air is immediately removed and drained out of the test system. Therefore, oxygen supply is always sufficient and measurement of the oxygen concentration is unnecessary.

The airflow or aerosol flow to each rat port was ~1 L/min, which is assumed to be laminar. The total flow rate through the test unit with 16 ports was ~40 L/min; the total volume of the inhalation system (excluding the mixing box) ensures that the intended concentration of the test item was reached shortly after start of exposure (50% concentration value after ~4 min). The test item was aerosolized using a dry dispersion system operated with pressurized air. The aerosol generator (TOPAS Co.) was provided by Technical University Dresden, Germany and had already been used in the preparation period at TU Dresden to characterize and optimize the physicochemical properties of the aerosol and to investigate aerosol altering, re-agglomeration and precipitation generated mass loss all over the test unit. The feasible limit concentration for non-altering aerosol concentration was determined using this equipment. Because the impact of equipment, hoses, hose material, hose length/diameter and other design parameters heavily influences aerosol behavior, the animal test set-up was designed to duplicate as closely as possible the TU Dresden (9) set-up to create valid aerosol generation. The system was tested without animals to demonstrate the test system compliance (9). This technical prework guaranteed that there were no aging effects on particle agglomerates in the 4 h exposure period of the animal experiment. An aerosol photometer signal was used to control the dispersion system feed rate to maintain a constant aerosol concentration in the inhalation unit. Actual test item concentrations were measured in the breathing zone of the animals.

### Monitoring and Controlling the Exposure Atmospheres

Air flow, temperature and relative humidity were measured continuously and recorded as 10-min means. The limits were set at 22 + 2°C for temperature and 55 + 15% for relative humidity. Animal room lighting was on a 12 h light/dark cycle controlled by an automatic timing device.

An aerosol photometer developed at Fraunhofer ITEM was used to continuously monitor the aerosol concentration. To adjust the photometer, the aerosol concentration was determined gravimetrically using filter samples (four times per 4 h inhalation).

The required MMAD range in acute toxicity studies is 1–4 μm. Due to the low test-item density, the aerosol showed rapid aging and a strong tendency to form large agglomerates out of this range. Thus, the experimental design given by the OECD TG needed adjustments (Table 1). The MMAD of the aerosol phase was determined by measuring the dry aerosol (optical particle sizers; two MMAD determinations were done within the 4 h exposure period).

The exposure set-up was documented by taking photographs. This included the formation and deposition of particle agglomerates in the interior conducting pipes of the exposure unit as well as external deposits in the animals' nasal areas (nostrils and whiskers).

### Animal Allocation and Treatment

Three young adult Wistar Crl:WI (Han) rats per sex (Charles River Deutschland, Sulzfeld, Germany), ~7 weeks of age at delivery, were allocated to this study. The animals were allowed to adjust and become acclimatized to the Fraunhofer ITEM environment for ~2 weeks. Animals were group-housed (separated by sex) in Makrolon® (polycarbonate) type IV cages. Cages and absorbing softwood bedding material (Lignocel BK8-15) were changed once weekly. Tap water from the Hannover city water supplier was offered fresh weekly in a Makrolon® bottle fitted with a stainless-steel nipple top with a hole ~0.5 mm in diameter. As diet, a commercial chow in pellet form was used, identified as ssniff V1534, purchased from ssniff-Spezialdiaeten GmbH (Soest, Germany). The diet was offered fresh weekly. The temperature and the relative humidity of the animal room were monitored electronically and recorded on a continuous basis. The limits were set at 22 + 2°C for temperature and 55 + 15% for relative humidity. A 12 h light/dark cycle was used controlled by an automatic timing device. The air exchange rate was at least 10 times per hour. Clinical observations were made during inhalation and, when inhalation was stopped ahead of schedule (~3 h after the start of the experiment), the three surviving animals were observed in their cages. Two of these animals died spontaneously after transfer to the cage and one animal was euthanized.

Body weights were measured during the acclimatization period (once; randomization weighing).

The rats were exposed to an aerosol generated by a flow-past, nose-only inhalation exposure system at Fraunhofer ITEM, Hannover, Germany. For 2 weeks before exposure, rats were trained to the exposure tubes avoiding undue stress on the animals. Animal restraining tubes are constructed in such a way that hyperthermic effects on rats cannot occur. In this system, the aerosol is supplied to each animal individually and exhaled air is drawn off immediately. The rats are placed around the exposure cylinder in tapered acrylic glass tubes with adjustable backstops. Historical measurements have confirmed that there are no differences in concentrations among the different outlets.

### Necropsy and Histological Processing

Necropsies were performed at Fraunhofer Institute for Toxicology and Experimental Medicine (ITEM), Hannover, Germany. The lung and the lower half of the trachea were weighed and used for histopathology. The lungs were inflated under a pressure of about 20 cm water column with formalin and fixed by immersion. An extensive set of organs and tissues (nasal cavities, larynx/laryngopharynx, trachea, lungs, heart, thoracic aorta, tongue, esophagus, salivary glands (mandibular, sublingual, parotic), gastrointestinal tract including Peyer's patches, liver, pancreas, thymus, spleen, lymph nodes (bronchial, mandibular, mediastinal, mesenteric), kidneys, urinary bladder, testes/epididymides, prostate, seminal vesicles, ovaries/uterus with cervix and vagina, thyroids with parathyroids, adrenals, pituitary gland, brain, spinal cord, sciatic nerve, eyes with optic nerves and Harderian glands, skin, mammary glands, skeletal muscle, sternum and femur with bone marrow) was sampled and subjected to macroscopic evaluation. The tissues were fixed in 10% neutral-buffered-formalin, the eyes with optic nerves and Harderian glands were fixed in Davidson's fixative and the nasal cavities were deep frozen with liquid nitrogen to avoid loss of SAS in the cavities from rinsing out with liquid fixing solution. All samples were transferred to AnaPath Services GmbH, Liestal, Switzerland, where histological processing, EDX analysis and histopathological evaluation (Oberbuchsiten, Switzerland) were performed.

The lungs were trimmed according to Ruehl-Fehlert et al. (14), Kittel et al. (15) and Morawietz et al. (16). The left lobe was split at the bifurcation with one part frozen and cryosectioned for EDX analysis. The larynxes were frozen, trimmed at three levels according to the previously cited RITA recommendations and cryosectioned. At each level, one section was stained with HE and one was used for EDX analysis. The tracheas were frozen, trimmed longitudinally and cryosectioned, with one section used for HE-staining and one for EDX analysis. The nasal cavities were transferred frozen to AnaPath Services GmbH. For the two deceased males and the deceased female that died during the inhalation procedure, the nasal cavities were sawn with a diamond blade according to trimming scheme of Young (17) and Kittel et al. (15) and dried for EDX analysis. For the remaining animals, the nasal cavities were submerged and fixed in 100% ethanol and embedded in methyl methacrylate resin before being sawn with a diamond blade according to the same trimming scheme for EDX analysis.

All other tissues were trimmed, dehydrated, embedded in paraffin wax, sectioned at an approximate thickness of 4–5 μm and stained with hematoxylin and eosin (HE) according to AnaPath Services GmbH SOPs. The microscopic tissue sections were quality-controlled under the light microscope before being transferred to the study pathologist.

### Histopathological Evaluation

Histological changes were described, wherever possible, according to distribution, severity and morphologic character. Severity scores were assigned on a scale of 1–5.

Grade 1, Minimal: This corresponds to a histopathologic change ranging from inconspicuous to barely noticeable but so minor, small, or infrequent as to warrant no more than the least assignable grade. For multifocal or diffusely distributed lesions, this grade was used for processes where < ~10% of the tissue in an average high- power field was involved.

Grade 2, Slight: This corresponds to a histopathologic change that is a noticeable but not a prominent feature of the tissue. For multifocal or diffusely distributed lesions, this grade was used for processes where between ~10 and 25% of the tissue in an average high-power field was involved.

Grade 3, Moderate: This corresponds to a histopathologic change that is a prominent but not a dominant feature of the tissue. For multifocal or diffusely distributed lesions, this grade was used for processes where between ~25 and 50% of the tissue in an average high-power field was involved.

Grade 4, Marked: This corresponds to a histopathologic change that is a dominant but not an overwhelming feature of the tissue. For multifocal or diffusely distributed lesions, this grade was used for processes where between ~50 and 95% of the tissue in an average high-power field was involved.

Grade 5, Severe: This corresponds to a histopathologic change that is an overwhelming feature of the tissue. For multifocal or diffusely distributed lesions, this grade was used for processes where more than ~95% of the tissue in an average high-power field was involved.

### EDX Analysis

Sections from frozen tissues were placed on silicon-free plastic slides (EMS plastic slides, Electron Microscopy Sciences), silver coated at 10 μm thickness and inserted into the Phenom^TM^ Pharos (Thermo^TM^ Scientific) SEM for scanning electron microscope imaging and energy dispersive X-ray (EDX) point analysis. The tissues were systematically searched and particles/spots with suspicious contrast or morphology were point analyzed. The analyses were conducted using the following specifications: mode 15 kV, intensity point, detector BSD Full and vacuum 60 Pa. The frozen nasal cavities were dried, sawn with a diamond blade, resin embedded and sawn again, then coated and analyzed using an identical procedure.

## Results

### Contact Angle Measurement of Test Item (Prüfbericht TU Dresden 2021)

The contact angle of 147.21 ± 5.95° revealed hydrophobic properties of the test item (Figure 1).

### Aerosol Characterization

Due to dead/moribund animals, the exposure had to be stopped after 3 h and surviving animals transferred to cages. Because of the shortened exposure period, only three gravimetrical filters were taken: 494.0, 519.8 and 537.9 mg/m^3^; mean 517.2 + 18.0 mg/m^3^ (*N* = 3).

Aerosol concentration was recorded on-line in parallel using an aerosol photometer (light scattering).

As is normal, the particle size distribution was measured at one port of the test system using a cascade impactor indicating at the time of measurement a MMAD of 1.31 μm. However, the pretest comparison, without rats, using laser diffraction and cascade compactor measurements for the test unit and test conditions over 4 h clearly demonstrated that the cascade impactor results always led to a much finer particle size distribution due to the induced shear forces on the agglomerated aerosols in the cascade impactor. Therefore, the MMAD results of the cascade impactor provided the wrong impression of fine particles while in reality the particles size though ongoing agglomeration was actually increasing over the time, more rapidly at the beginning of the test but dynamically, all over the time. Details are provided by Wessely et al. (9). Cascade impactor measurements subpress the aerosol altering through re-dispersion, as part of the measurement systematic and thus cannot used as quality criteria for fluffy powders with low tapped density.

### In-Life Observations Including Inhalation and Post-Inhalation Period

The first male was found dead after 2$\frac{3}{4}$ h of exposure, 5 min after showing a reduced respiratory rate, followed by one male and one female 3 h after the start of exposure, prior to death, the rats showed signs of anemia and a reduced respiratory rate. Because of these mortalities, the exposure was stopped after 3$\frac{1}{4}$ h for animal welfare reasons and the remaining male and two females were transferred to their cages. One hour later, one further male and female with respiratory distress were found dead. The last animal was sacrificed for concurrent moribund conditions and respiratory distress.

### Gross Lesions

**Figure 2:**
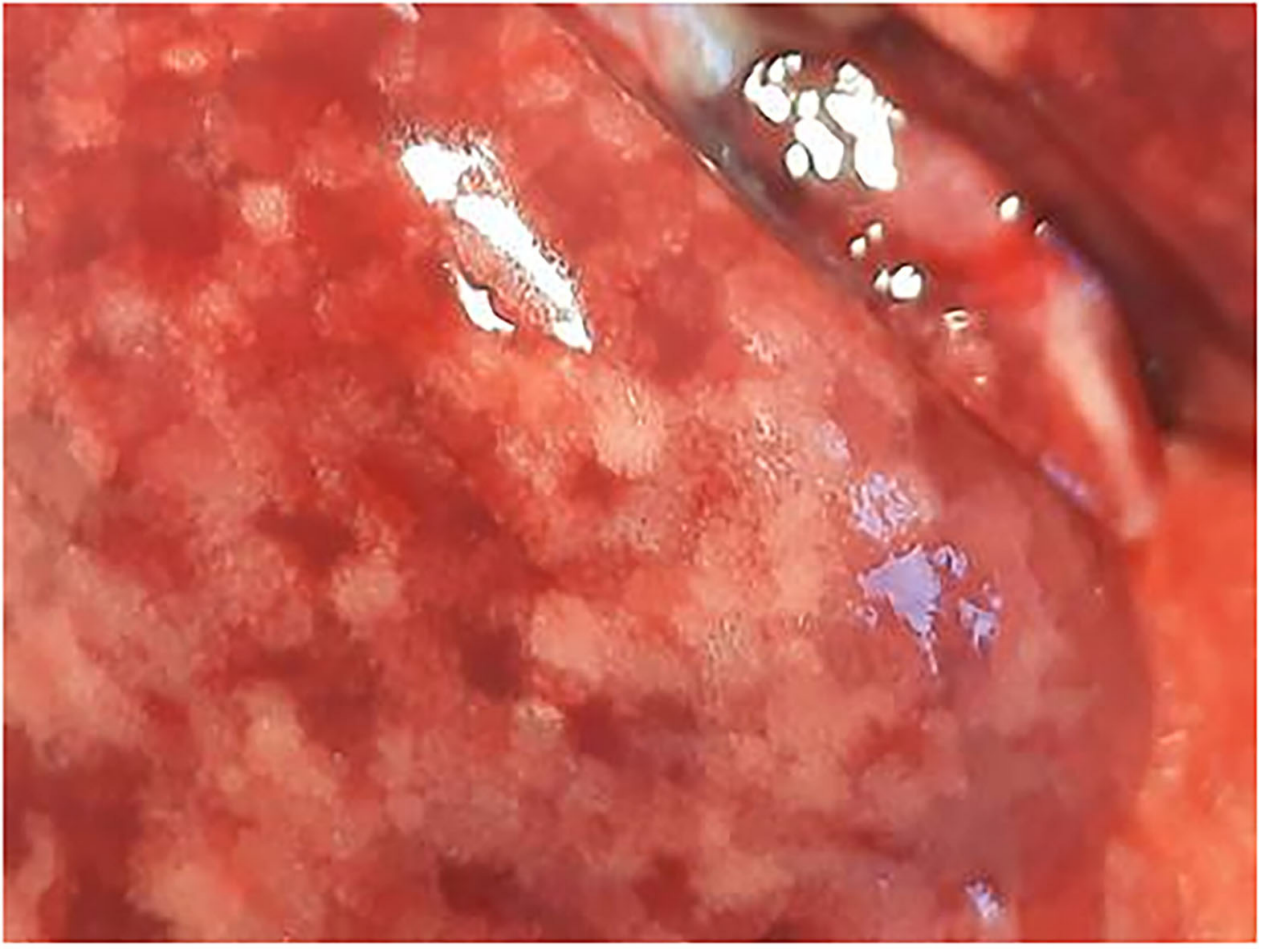
Male rat. Deceased 1 h. after inhalation (in cage). Marbled discoloration of lung surface due to congestion. Visible focal petechia. Whitish areas are representative of air-filled alveolar structures (indicative of acute emphysema).

The animals underwent necropsy within 10 min after death. No external deposits of test material were observed in the animals' external nasal areas (nostrils and whiskers). The lungs of all animals were discolored dark red or spotted and showed a spongy consistency (Figure 2). These findings were consistent with congestion, edema, acute emphysema and petechiae. In the trachea of the deceased animals there was a foamy content.

### Histopathology and EDX Evaluation Nasal Cavities

**Figure 3:**
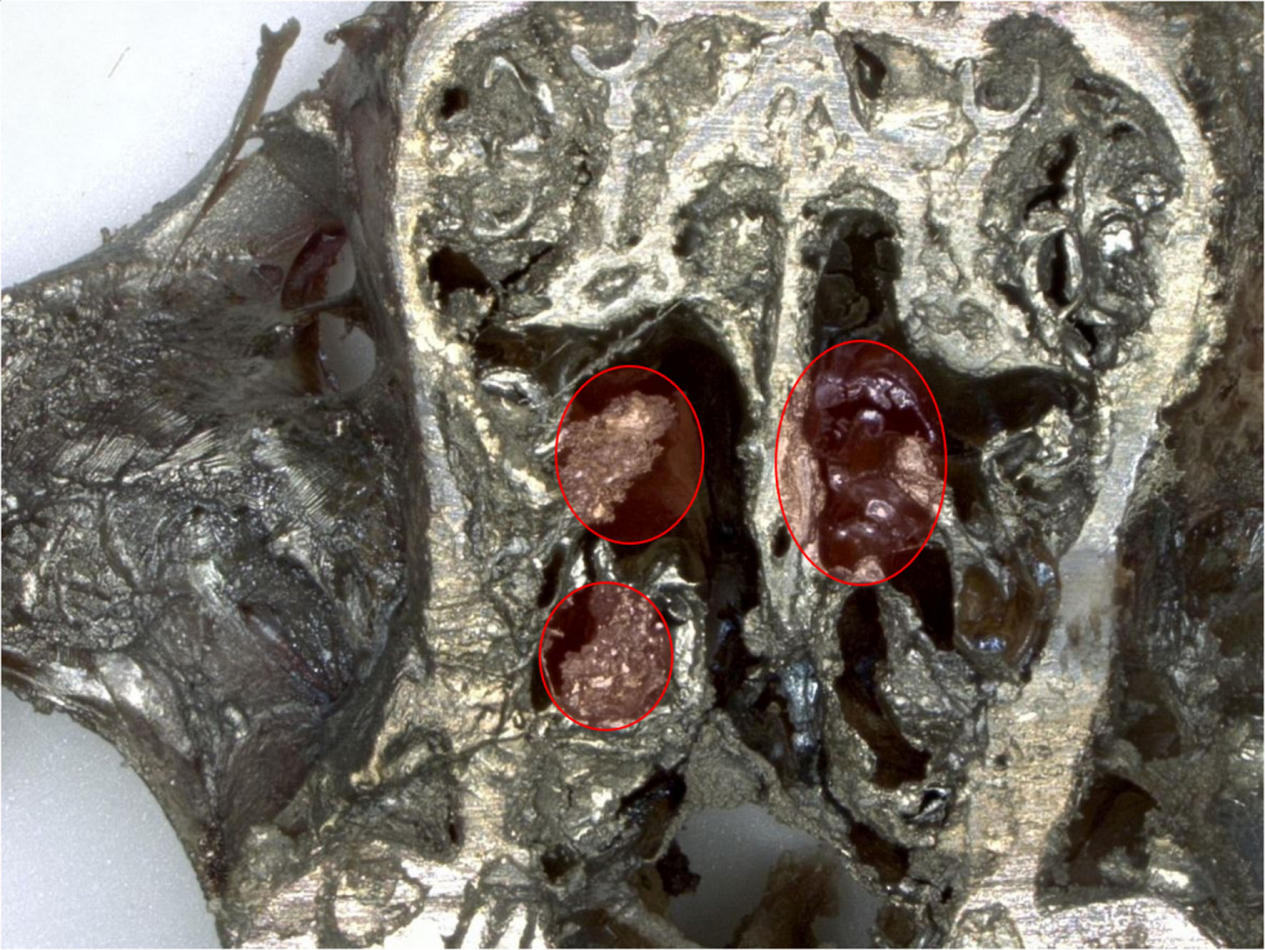
Male rat. Deceased after 2$\frac{3}{4}$ h after start of inhalation. Nasal cavity level 4 (frozen, dried, sputtered sample): partial blockage by deposited test item. Digital microscopy, lens x30.

**Figure 4:**
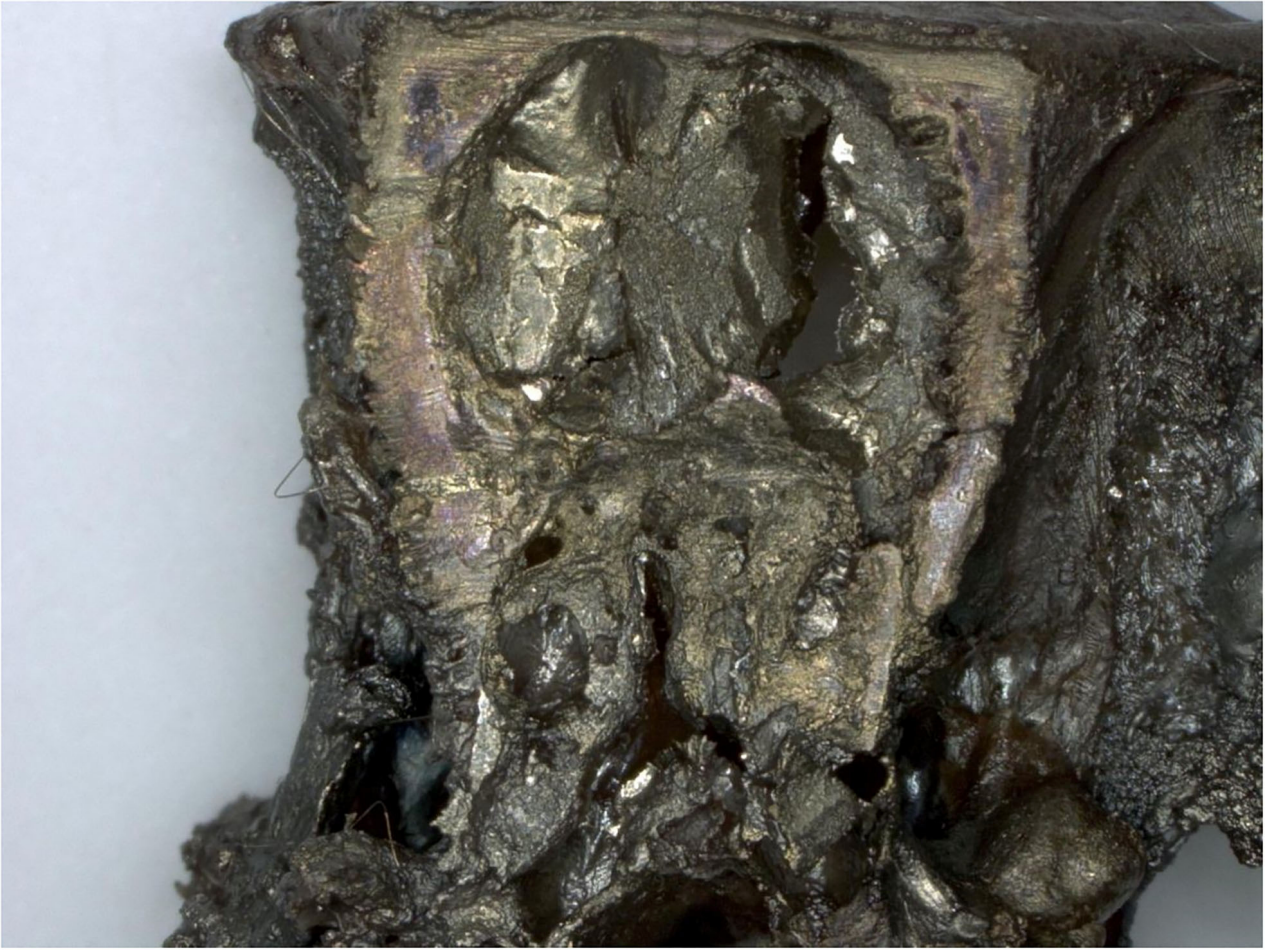
Female rat. Deceased 1 h. after transfer to cage. Nasal cavity level 4 (frozen, dried, sputtered sample): complete blockage by deposited test item. Digital microscopy, lens x30.

**Figure 5:**
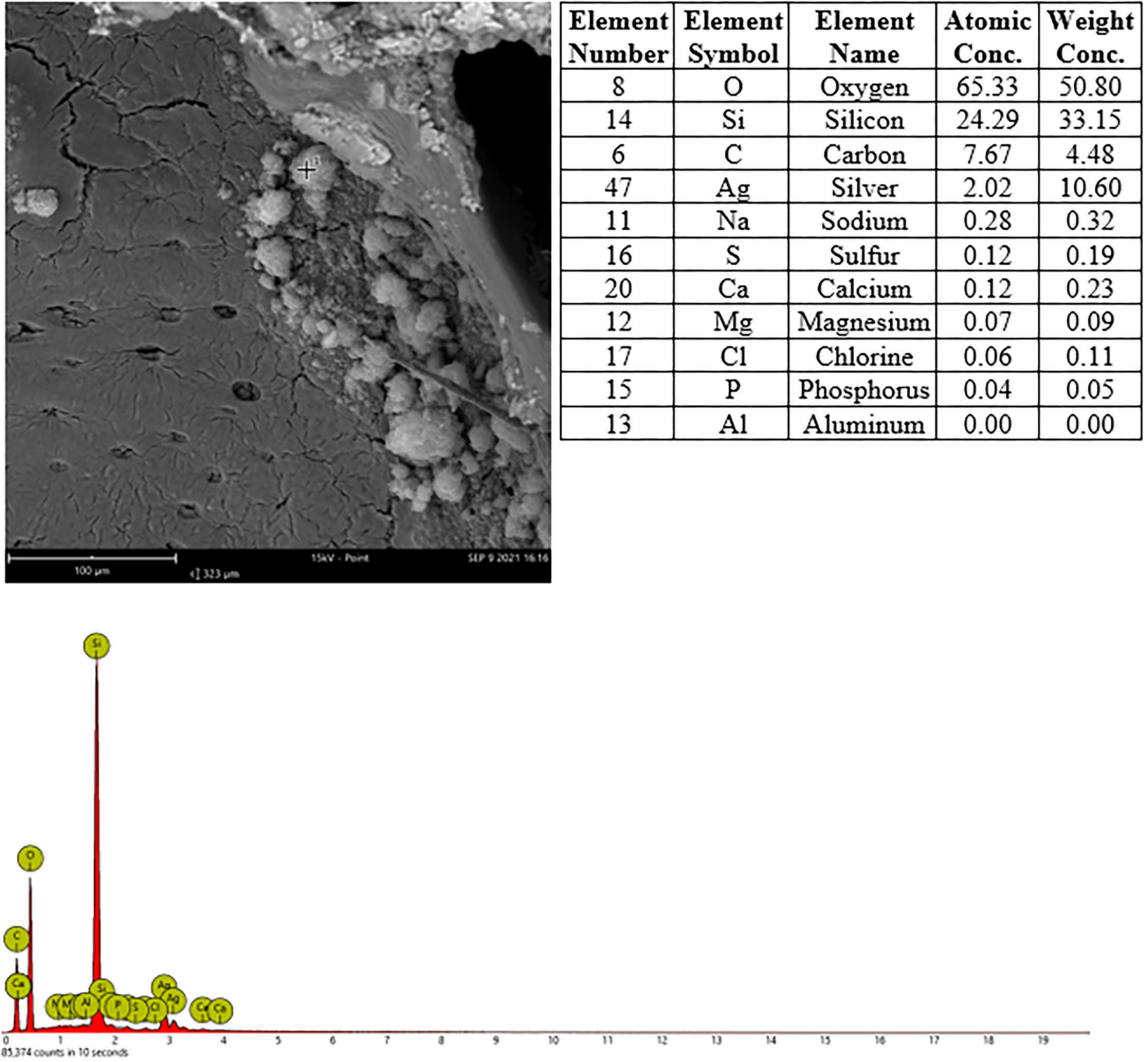
Male rat. Deceased after 2$\frac{3}{4}$ h after start of inhalation. Nasal cavity, frozen and dried, level 3, area 3, EDX point analysis showing SEM image, EDX spectrum, and atomic and weight concentration table of detected Si. Note: agglomeration of particles.

**Figure 6:**
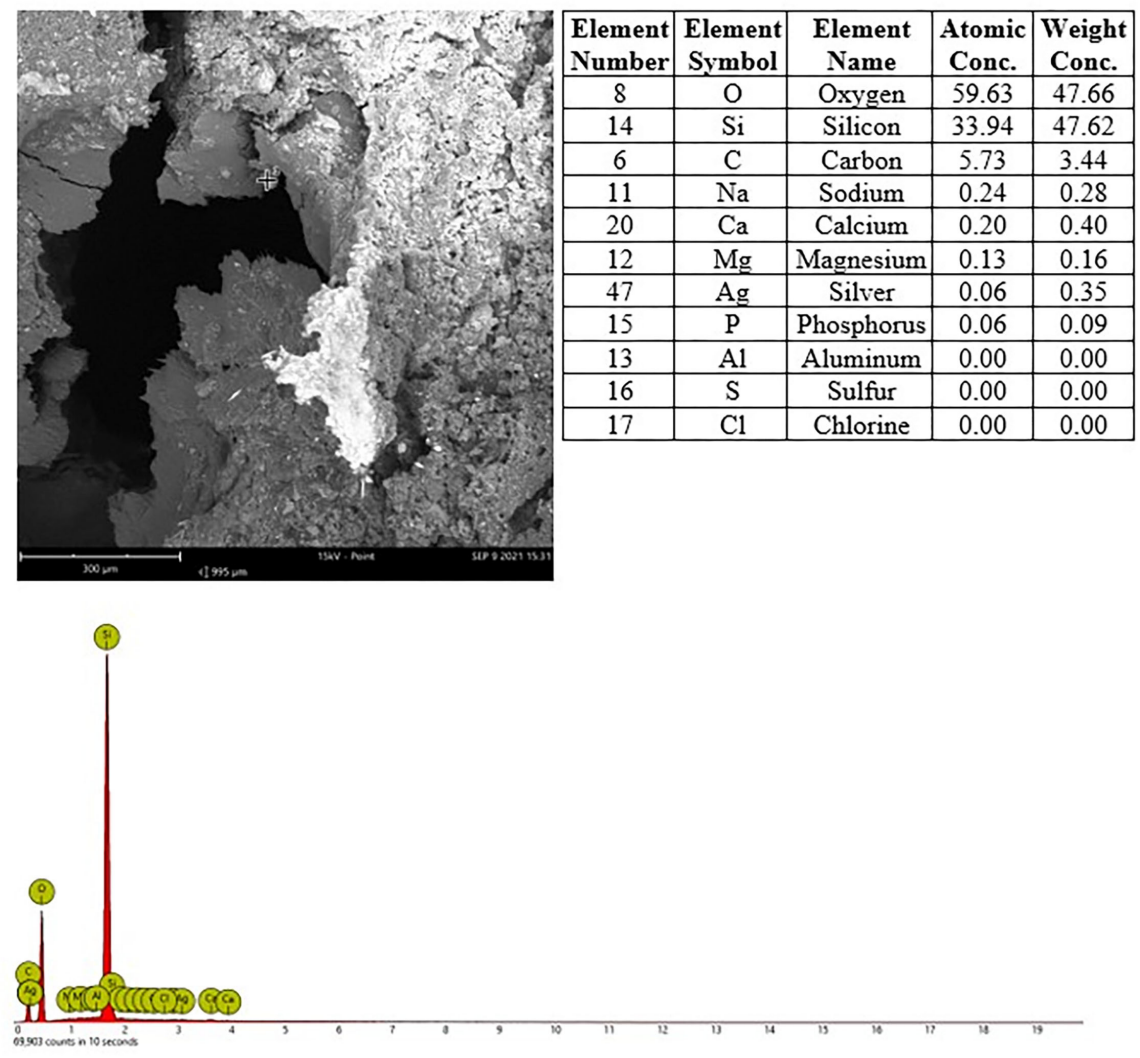
Male rat. Deceased after 3$\frac{1}{4}$ h after start of inhalation, Nasal cavity, frozen and dried, level 4, area 2, EDX point analysis showing SEM image, EDX spectrum, and atomic and weight concentration table of detected Si.

In nasal cavities processed from frozen material and sputtered for subsequent EDX analysis, the evaluation under digital microscopy revealed deposition of foreign material in all nasal cavity at levels 3 and 4. Only a minimal deposition was noted in nasal cavity at level 3 (Figure 5). In contrast there was partial to almost complete blockage by foreign material deposition in nasal cavity level 4 (Figures 3, 4). By SEM-EDX evaluation, the test item was found accumulated as larger SAS particles with a more gel-like appearance (Figures 5, 6).

### Larynx, Trachea With Bronchial Bifurcation and Carina

Histologically, there were no findings in larynx, trachea, bronchial bifurcation or carina. By EDX, minor amounts of Si were detected in the larynx and trachea of only one animal, the small speck detected in the trachea was deemed to be from background contamination.

### Lungs

**Figure 7:**
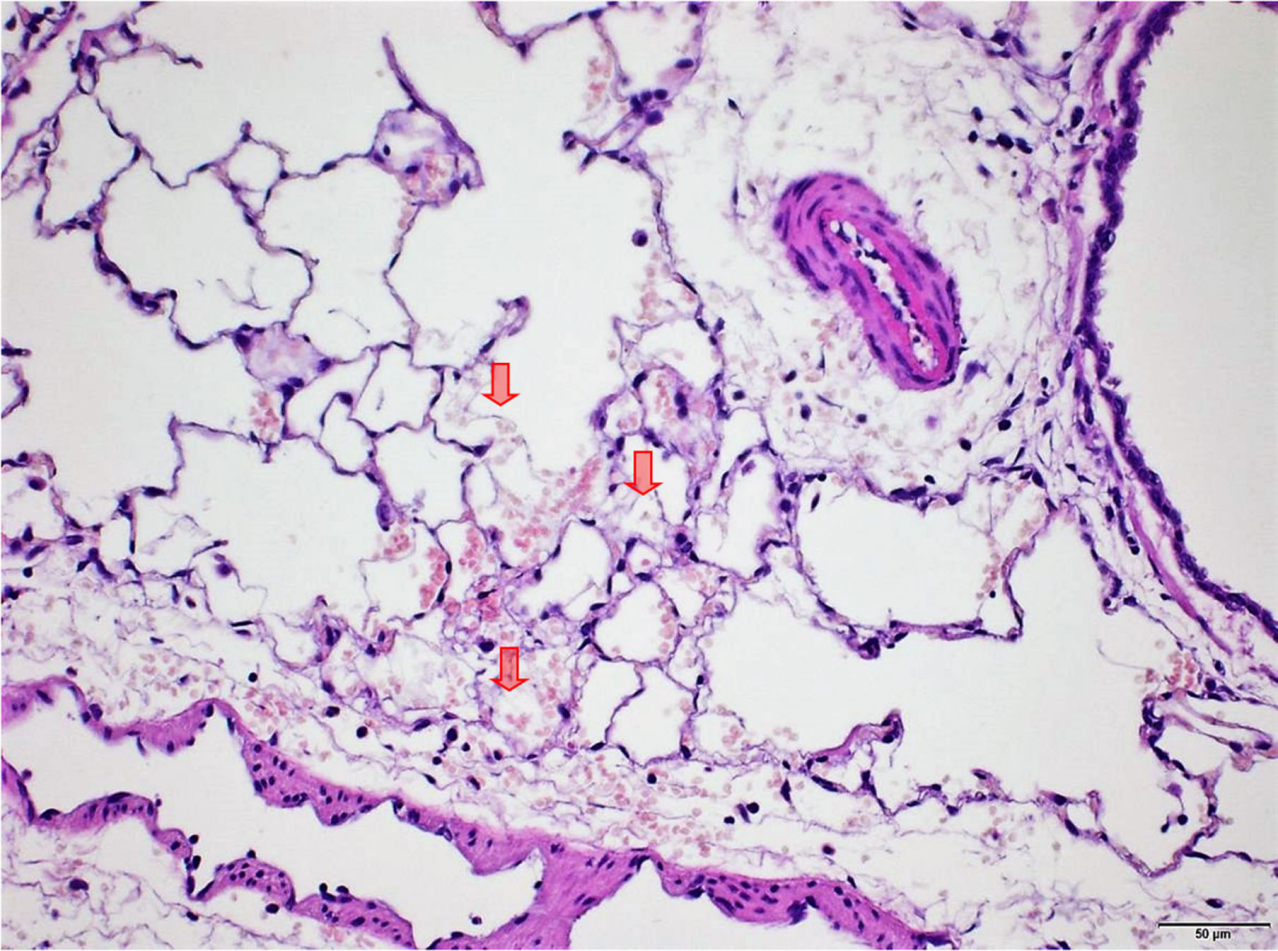
Male rat. Deceased 2$\frac{3}{4}$ h after inhalation start. Lung, right caudal lobe. Note fibrin in alveoli (arrows) associated with tiny hemorrhagic foci. HE, original magnification x20.

**Figure 8:**
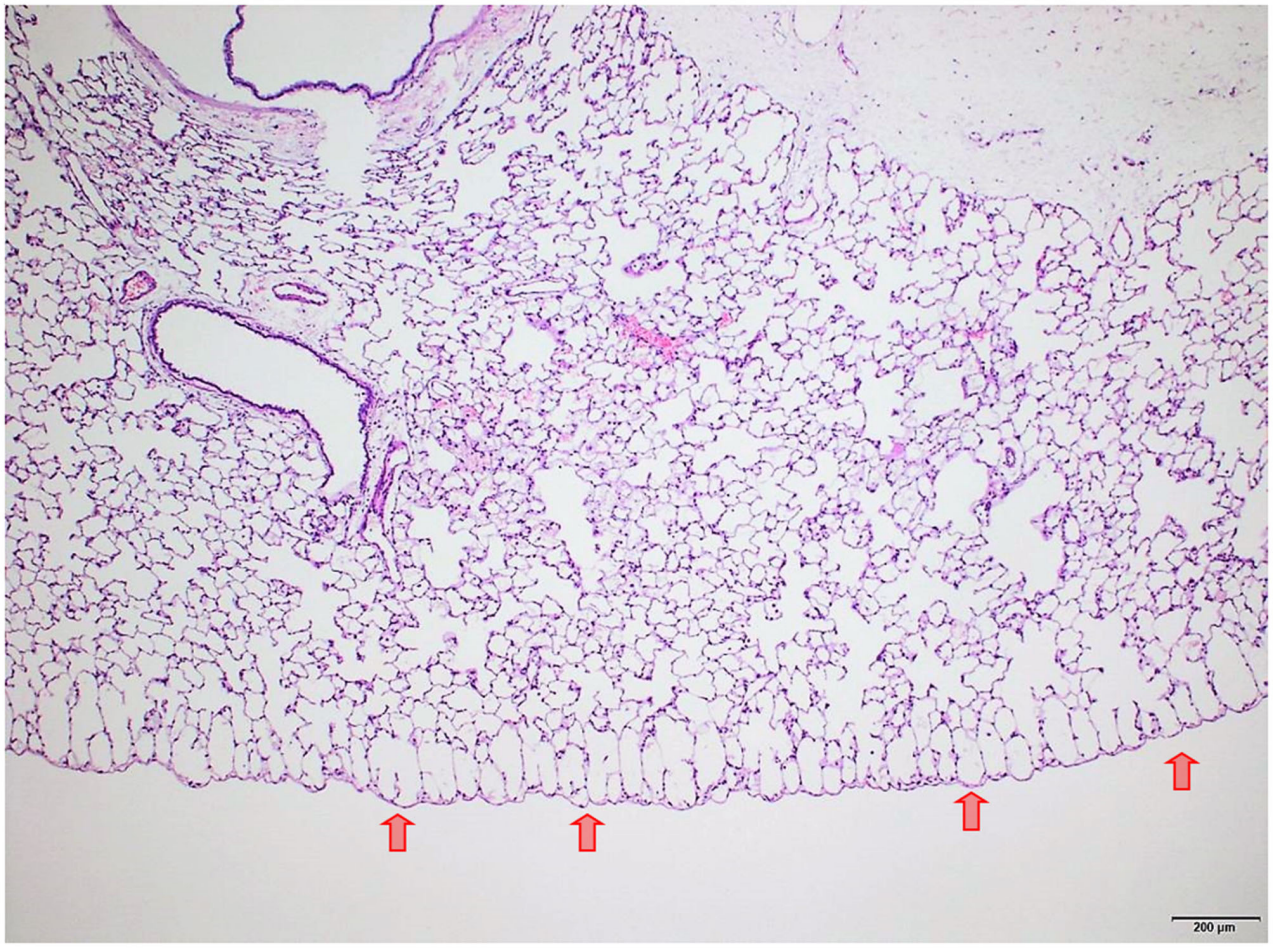
Male rat. Deceased 3$\frac{1}{4}$ h after inhalation start. Lung, left lobe. Focal subpleural acute emphysema (arrows). HE, original magnification x20.

**Figure 9:**
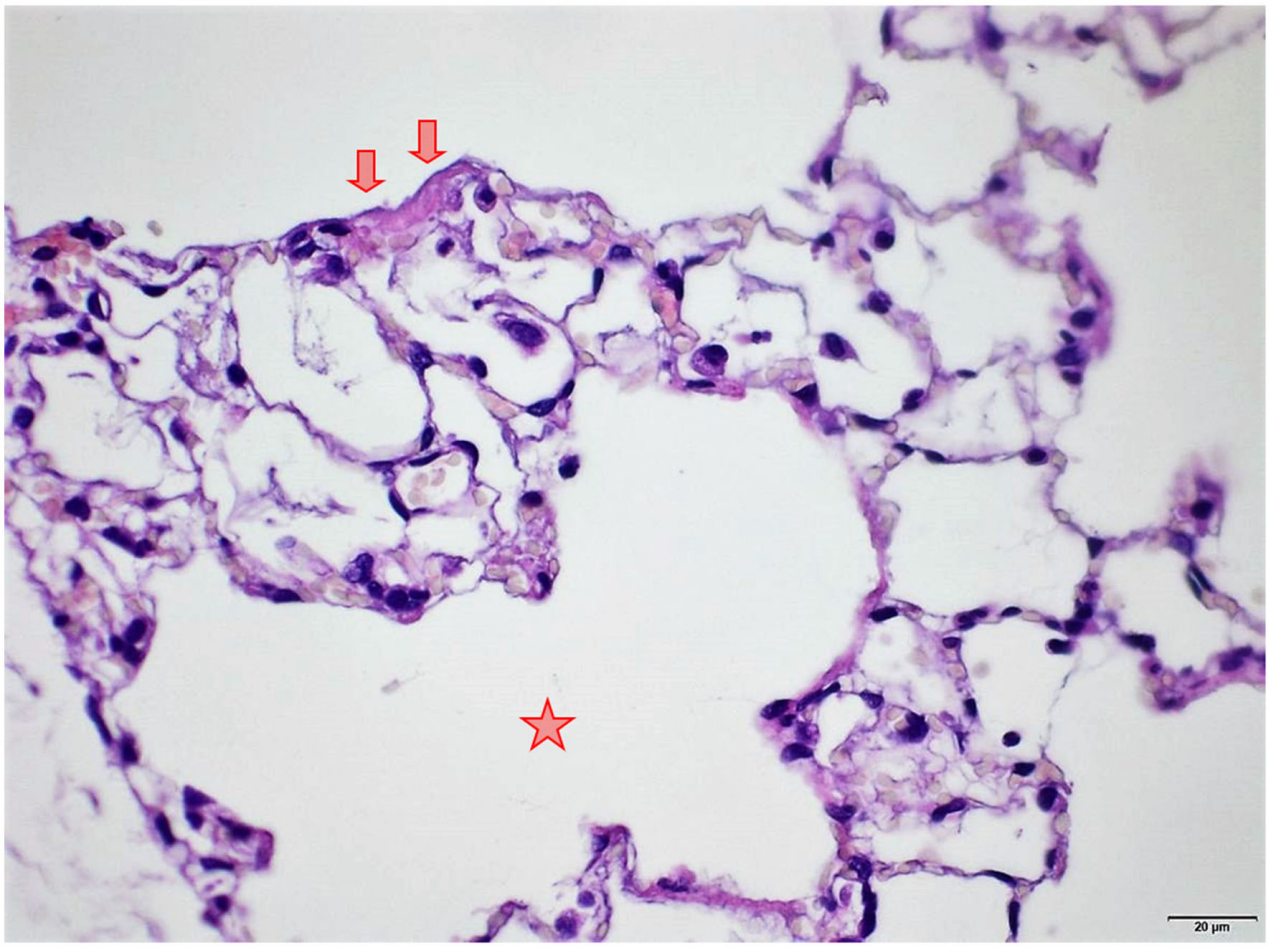
Female rat. Deceased 1 h. after transfer to cage. Lung, right middle lobe. Focal acute emphysema (asterisk) and focal alveolar wall necrosis (arrow). HE, original magnification x20.

Several findings were noted in lungs that are related to respiratory failure, including focal to multifocal hemorrhages (Figure 7) representing petechiae, alveolar fibrin (Figure 7), focal to multifocal acute emphysema (Figures 8, 9) and, rarely, focal alveolar wall necrosis (Figure 9). Subsequent mixed-cell infiltrate and macrophages were noted in several samples (Table 2). No Si was detected in lungs by EDX.

**Table 2:** Findings in lungs separated per lung lobe.

**Lung Lobe**	**Left**		**Right Cranial**		**Right Medial**		**Right Caudal**		**Accessory**	
**No. of samples/sex**	**(18) M**	**(18) F**	**(18) M**	**(18) F**	**(18) M**	**(18) F**	**(18) M**	**(18) F**	**(18) M**	**(18) F**
**No. affected/mean severity**										
Alveolar hemorrhage	2/1.5	2/1.0	1/1.0	0	1/1.0	1/1.0	2/1.5	2/1.0	0	1/1.0
Fibrin, alveolar	2/1.0	2/1.0	2/1.0	0	2/1.0	1/1.0	2/1.5	3/1.0	1/1.0	0
Alveolar macrophages	2/1.0	2/1.0	1/1.0	0	2/1.0	0	1/1.0	1/1.0	1/1.0	0
Infiltrate, mixed cell	1/1.0	0	1/1.0	0	1/1.0	1/1.0	0	1/1.0	1/1.0	1/1.0
Emphysema, acute	2/1.0	1/1.0	1/1.0	0	3/1.3	3/1.0	1/1.0	3/1.0	0	2/1.5
Alveolar wall necrosis	0	0	0	0	0	1/1.0	1/1.0	1/1.0	0	0

### Other Organs

In four animals (two per sex), there were focal to multifocal hemorrhages in the thymus. No findings in the remaining organs could be attributed to treatment or as the cause of morbidity/death.

## Discussion

The European Risk Assessment Committee (RAC) proposed the classification for hydrophobic HMDZ surface-treated SAS as Acute Tox Cat 2 (fatal if inhaled) based on the lethality to test animals observed in an acute inhalation study performed in Wistar rats with a calculated 4 h LC50 of 450 mg/m^3^ (18). Re-evaluating these data, it was assumed that no conclusion could be drawn as to the cause of lethality in the seven of 10 animals that died at 540 mg/m^3^ during the study based on macroscopical examination of the outer surfaces of the organs in the abdominal and thoracal cavity only. Although clinical effects of respiratory distress and macroscopical findings, such as lumps of white particles and slime in the nose and focal hemorrhages on the lung surface representing petechiae, are described as indicators of suffocation, no definite proof of physical obstruction is given in these existing studies. Other unpublished studies, which were conducted in the same decade showed no mortality or mortality at other concentrations but the reports contained even less information. While conformity with guidelines was cited in all these studies, the scientific protocol is considered inadequate to answer the cause of mortality observed in these studies. Because the investigations required to address the exact cause of lethality in connection with particles was not considered in these previous existing studies, a new acute inhalation study with an extended design was conducted. As a first step, a dry run was performed without animals for technical optimization. This consisted of monitoring and detailed characterization of the aerosol in the test equipment (i.e., MMAD stability, particle concentration, particle size distribution over time, aging effect) at various sites of the exposure tower, focusing on the point of aerosol generation and the main outlet port. Overall, it was shown that in a concentration range of 500–600 mg/m^3^ it was feasible to generate an atmosphere that fulfilled the OECD 436 requirement of MMAD stability over 4 h [within 1–4 μm range (9)].

The TU Dresden investigation and the dry run with the Fraunhofer equipment set-up which was later used, without modification, for the animal study clearly demonstrated that the test aerosol atmosphere at a concentration of 500 to 600 mg/m^3^ is sufficiently stable and remains within the required MMAD window at the point of exposure. The starting concentration in this study was chosen as 500 mg/m^3^ hydrophobic HMDZ surface-treated SAS for 4 h based on the results provided by TU Dresden regarding still acceptable aerosol alteration in the test unit in line with the OECD 436 requirements. The aerosol was supplied to the rats by a flow-past, nose-only inhalation exposure system at Fraunhofer ITEM, Hannover, Germany. This ensured the highest standards in having the same test atmosphere in each animal's breathing zone. Three young adult Wistar Crl:WI (Han) rats per sex were allocated to this study. Five animals died spontaneously during the study, and one animal was euthanized for animal welfare reasons. All these animals showed respiratory distress, such as preterminal gasping and a reduced respiratory rate prior to death. A few animals appeared to be anemic.

The working hypothesis was that a single inhalation of this high concentration may cause a physical obstruction of the rat upper respiratory tract and that mechanical blockage of the respiratory tract associated with suffocation as cause of mortality would be made evident by histopathological examination, especially when the upper respiratory tract is included. The animals underwent an immediate necropsy (within 10 min. after death to avoid autolysis) and an extended list of organs and tissues was sampled according to an optimized protocol that ensures preservation of the organs, including deposits. The fixed tissues underwent histological and EDX evaluation. Tissues taken from nasal cavities, parts of the lungs, trachea and larynx were initially frozen to avoid loss of SAS in the cavities from rinsing out with liquid fixing solution.

In nasal cavities processed from frozen material and sputtered for subsequent EDX analysis, the evaluation under digital microscopy revealed deposition of foreign material in all nasal cavity at levels 3 and 4. Only minimal deposition was noted in nasal cavity level 3. However, there was almost complete blockage by foreign material deposition in nasal cavity level 4. By SEM-EDX evaluation, the material was confirmed as the test item by the presence of Si. Rats are obligatory nose breathers (19) and sudden death by deposition of material in the nasal cavities, e.g., reflux (20, 21) has been reported. EDX could only be performed on frozen tissues from the three sampled rats, i.e., resin embedded nasal cavities from the other three animals revealed no results. Note that the EDX beam can only penetrate ~2 μm (of solid carbon), therefore, only Si within this depth could have been detected. However, the Si in nasal cavity samples was found at deeper levels; the frozen, dried, and sawed (Si-free diamond blade) nasal cavities showed remarkably large areas of accumulated Si in all three animals at nasal cavity levels 3 and/or 4. Aside from normal tissue elements Ag from the sputtering and Cl from the plastic slide section were also detected. The visible test item morphology detected in the frozen samples was observed to have changed into larger particulate structures with more gel-like appearance.

During necropsy, the lungs of all animals were described as dark red with a sponge-like consistency or as dark red and spotted. The reason for these changes was macroscopically visible as congestion, edema, acute emphysema and petechiae. Histologically, there were focal to multifocal hemorrhages, alveolar fibrin, focal to multifocal acute emphysema, and, occasionally focal alveolar wall necrosis associated in some samples with subsequent mixed-cell infiltrate and macrophages. All these findings are associated with respiratory failure, i.e., mechanical asphyxia (22). Test item (Si) was not detected in the lungs by EDX. The lesions in lungs are typical of suffocation, i.e., “*Pulmonary edema with or without hemorrhage, atelectasis and interstitial emphysema are common macroscopic and microscopic lesions in animal and human victims*.”. In obstructive asphyxia, there is immediate dyspnea with convulsions, followed by bradycardia and apnea, an isoelectric EEG, agonal respirations, and cardiac arrest usually within 4 to 6 min (23).

In four animals (two animals per sex), there were focal or multifocal hemorrhages representing petechiae. Petechiae are not definitive proof but rather, a feature supporting asphyxia (22, 24).

Foamy content in the trachea of the animals was described during necropsy and is considered to be a consequence of asphyxia associated with preterminal gasping and edema. A spot with Si was only found in one animal and the larynx of another animal was found to contain Si, however, no deposit was noted histologically. This is due to the loss of the test item from water solubility during processing. Histologically, there were no findings in the larynx, trachea, bronchial bifurcation or carina. Settlement of the test item in the larynx of one animal may be considered supportive of asphyxia caused by obstruction of the nasal cavities as demonstrated in this study.

However, it is not possible to determine exactly at which concentration the physical obstruction of the nasal cavity for the respective hydrophobic particulate substance occurs, e.g., some hydrophobic organic pigments show 4 h LC50-value above 1,000 mg/m^3^ (12). In this study, mortality is discussed as a consequence of the limited wettability of the test material. The contact angle is a possible way to express the wettability and to predict the test material behavior. A contact angle of 147.21 ± 5.95° represents a very reduced wettability. Therefore, a re-agglomeration of particles through a physical effect is highly probable. It is known from investigations of the contact angle of different organic pigments that the higher the contact angle, the higher the mortality in acute rat studies; whereas, hydrophilic particles with a lower contact angle are unlikely to cause death by asphyxiation due to their low re-agglomeration potential (12). When characterizing the test material in the present study, the contact angle was also measured. The results show that it is extremely difficult to apply a defined water droplet on the surface of the test material because the droplet can move easily due to the lack of interaction with the test material, resulting in a contact angle above 140°. It can be concluded that in the present case no inhalation test equipment design would lead to different results, i.e., the study outcome depends on the physicochemical properties of the test item. Hofmann et al. (12) showed that contact angle measurements resulting in angles demonstrating hydrophobicity are considered indicative of animal study outcome, with high hydrophobic particle concentration leading to lethality in rats. Hydrophobicity is another physicochemical property of HMDZ surface treated SAS which, in combination with the low density of the large light and fluffy agglomerates, leads more readily to upper respiratory tract blockage. The mechanism causing lethality of hydrophobic particles by mechanical obstruction of nasal cavities was convincingly demonstrated in this study, Determining exact respective 4 h LC50 values would require numerous animal testing for each hydrophobic material. However, the scientific value of 4 h LC 50 inhalation studies to determine the concentration leading to physical obstruction and suffocation is highly questionable. This also applies to repeated testing in subacute or subchronic animal inhalation studies to detect nasal cavity obstructions for regulatory purposes, This is also totally unjustifiable from an animal welfare point of view.

## Conclusion

The observation of lethality with low density surface treated hydrophobic SAS in acute high dose inhalation studies with rat led to wrong interpretations when toxicological assumptions were derived from this. We have clearly shown that the lethal effect in rat was caused by mechanical obstruction of the upper respiratory tract based on physicochemical properties of the inhaled particles. Based on differences in respiration and respiratory tract (upper and lower) anatomy, results from rats cannot be transferred to humans. In contrast to rats, humans are capable of breathing through the nose and mouth unlike the rat which is an obligatory nose breather. The scientific value of acute or repeated dose animal studies for all REACH registrations of hydrophobic particles to determine the exact concentration causing airway obstruction in the upper respiratory tract is not justifiable when considering animal welfare and it is also unnecessary from a regulatory point of view as OECD guidance 39 paragraph 51 (OECD, 2009) expressly points out that physical obstruction should not be misdiagnosed as a toxic effect. Physical obstruction of the nasal cavities in the obligatory nose-breathing rat occurs at an SAS concentration of 450 mg/m^3^ (18), suggesting that the numeric cut-off criteria for acute inhalation toxicity study for CLP classifications, are too high for hydrophobic low density powder particles and are not reflective of what could happen in humans.

The assumption that an exposure to concentrations of 450 mg/m^3^ and even greater in the rat experiments can allow statements on the toxicological effect of the substance seems very inappropriate A hazard classification for hydrophobic surface treated SAS particles, based on physical obstruction of the rat upper respiratory tract is thus unwarranted.

## Data Availability Statement

The original contributions presented in the study are included in the article/supplementary material, further inquiries can be directed to the corresponding author/s.

## Author Contributions

All authors listed have made a substantial, direct, and intellectual contribution to the work, and approved it for publication.

## Conflict of Interest

NK and TS are employed by Evonik Operations GmbH. KW, NW, and AV are employed by AnaPath Services GmbH. JN is employed by Grace Europe Holding GmbH. GG and OC are employed by Fraunhofer Institute for Toxicology and Experimental Medicine. BW and MS are employed by Technische Universität Dresden. VM is employed by Cabot Corporation. MK is employed by Wacker Chemie AG.

## Publisher's Note

All claims expressed in this article are solely those of the authors and do not necessarily represent those of their affiliated organizations, or those of the publisher, the editors and the reviewers. Any product that may be evaluated in this article, or claim that may be made by its manufacturer, is not guaranteed or endorsed by the publisher.
